# Immunization With Recombinant *Haemonchus contortus* Y75B8A.8 Partially Protects Local Crossbred Female Goats From *Haemonchus contortus* Infection

**DOI:** 10.3389/fvets.2022.765700

**Published:** 2022-04-04

**Authors:** Xiaowei Tian, Mingmin Lu, Yongqian Bu, Yang Zhang, Kalibixiati Aimulajiang, Meng Liang, Charles Li, Ruofeng Yan, Lixin Xu, Xiaokai Song, Xiangrui Li

**Affiliations:** ^1^Xinxiang Key Laboratory of Pathogenic Biology, School of Basic Medical Sciences, Xinxiang Medical University, Xinxiang, China; ^2^MOE Joint International Research Laboratory of Animal Health and Food Safety, College of Veterinary Medicine, Nanjing Agricultural University, Nanjing, China; ^3^Animal Biosciences and Biotechnology Laboratory, Beltsville Agricultural Research Center, Agricultural Research Service, U.S. Department of Agriculture, Beltsville, MD, United States

**Keywords:** *Haemonchus contortus*, Hc8, passive immunization, active immunization, goats, recombinant vaccine

## Abstract

*Haemonchus contortus* Y75B8A.8 (Hc8) derived from *H. contortus* excretory–secretory (ES) products was identified as a functional inhibitor of goat interleukin 2 (IL-2). It may act as a vaccine candidate for the development of therapeutic strategies against *H. contortus* infection. In this research, recombinant Hc8 (rHc8) and goat anti-rHc8 polyclonal antibodies were employed to evaluate the protective capacities of Hc8 antigen against *H. contortus* infections *via* active and passive immunization trials, respectively. In both trials, local crossbred female goats aged 9–12 months old were randomly divided into three groups, five in each group, respectively. Parasitological examinations, including fecal egg counts (FEC), cumulative FEC (cFEC), and worm burdens, were performed. In addition, antibody levels in mucosal homogenate (MH) samples and hematological and immunological parameters were detected. In the passive trial, goats were intravenously immunized with 5 mg total IgG containing anti-rHc8 goat polyclonal antibodies. After twice immunization, compared with the challenged control group, cFEC was reduced by 39%. In addition, there was a 46% reduction of worm burdens compared with the challenged controls. In the active immunization trials, 500 μg of recombinant Hc8 protein was given subcutaneously twice to 9–12-month-old local crossbred female goats with a 2-week interval, resulting in the generation of high levels of antigen-specific circulating antibodies. Besides, cFEC and abomasal worm burden were reduced by 70 and 55%, respectively, compared with the challenged control group. In addition, immunized goats had higher mucosal homogenate IgA and hemoglobin levels than the challenged controls in both passive and active immunization trials. These preliminary results demonstrated the immunoprophylactic effects of Hc8 antigen and will inform new studies on ES proteins in developing subunit recombinant vaccines against *H. contortus*.

## Introduction

Haemonchosis, caused by the gastrointestinal nematode *Haemonchus contortus*, is a highly pathogenic disease with global distribution (1). Small ruminants are susceptible to *H. contortus*, which is responsible for causing anemia, emaciation, edema, and even death (especially in lambs) (2). So far, anthelmintic drugs are commonly employed to control the infection of *H. contortus*, resulting in the emergence of drug-resistant *H. contortus* strains. Moreover, frequent use of anthelmintics (e.g., albendazole and levamisole) poses a significant threat to the environment and food safety (3–6). Therefore, practical and feasible strategies are required to address this public health and food safety problem. Immune prevention like vaccination can be an effective measure that could be taken to prevent the infection of *H. contortus*. In 2014, Barbervax®, extracted from *H. contortus*, was authorized as a commercial vaccine by the Australian Pesticide and Veterinary Medicines Authority. However, the vaccine requires multiple vaccination times to offer adequate protection and needs to be produced in a cost-effective, reproducible, and safe manner (7). Therefore, developing viable alternative vaccines like recombinant subunit vaccines for controlling *H. contortus* infections is still challenging.

*H. contortus* excretory–secretory (ES) products (HcESPs) are considered to be the most immunogenic antigens, which have immunomodulatory effects on host immune responses (8–14). Researchers have made consistent efforts to investigate the abilities of HcESPs and their components in protecting hosts and understand their protective mechanism of action. Schallig et al. found that 15 and 24 kDa antigens from HcESPs could provide protective immunity against haemonchosis, resulting in significant reductions in mean fecal egg counts (FEC, 77%) and abomasal worm burden (85%) (15). Bu et al. demonstrated that rHcftt-2 identified from HcESPs reduced mean eggs per gram in feces (EPG) and worm burdens by 26.46 and 32.33%, respectively (16). More recently, *H. contortus* α/β-hydrolase domain protein (HcABHD) was identified as an immunomodulatory antigen that interacts with goat T cells, and it could induce significant reductions in egg shedding (54%) and worm burden (74.2%) (17). Given the performance of ES antigens for their immunogenicity and performance in recombinant forms, it was meaningful to explore more new ES proteins and their active form in vaccine development.

Like other intestinal helminths, host cellular immunity against *H. contortus* infection is associated with the establishment of type 2 immune response characterized by the secretion of interleukin (IL)-4, IL-5, and IL-13, and the development of a Th1-type immune response related to chronic infections (7, 18, 19). Although Th2-mediated mechanisms for intestinal worm expulsion are well-established, the immune mechanisms behind susceptibility to chronic infections are poorly understood. Notably, the establishment of chronic intestinal helminth infections could also be caused by the initiation of type 1 responses (19). Thus, in this regard, we aimed to identify the inhibitor of IL-2 that might be responsible for the regulation of host Th1 response in chronic *H. contortus* infections *in vitro*. In our preliminary study, *H. contortus* Y75B8A.8 (Hc8) (NCBI Database ID: gi|560120149) derived from HcESPs was identified as an inhibitor of goat interleukin 2 (IL-2), which could not only bind to goat recombinant IL-2 (rIL-2) but also significantly inhibit the biological activity of rIL-2 *in vitro* (20). Besides, Hc8 contains a conserved domain Med15, and the latter was described as a critical transducer of gene activation signals in controlling early metazoan development (21). In addition, as a member of HcESPs, Hc8 may serve as a vaccine candidate for prophylactic intervention. Hence, we hereby aimed to validate its immune protective roles against *H. contortus* infection. Active immunization could confer long-term protection, whereas the protection offered by passive immunization lasts for a few weeks or months. Both ways of gaining immunity, either from active or passive immunization, can be used for disease control and prevention. Thus, we investigated the efficacies of the recombinant version of Hc8, and its specific antibody in the setting of laboratory trials *via* the analysis of host immune response after immunization.

## Materials and Methods

### Animals and Parasites

Local crossbred female goats (9–12 months old) purchased from a local company (Prosperous Sheep Industry, Nantong, China) were maintained indoor in helminth-free cages individually at Nanjing Agricultural University Experimental Animal Center prior to the experiments. Upon acquisition, FEC were carried out immediately to evaluate the health status of the goats, and no coccidian oocytes or nematode/cestode eggs were observed in their fecal samples. Experimental goats were daily provided with granulated feed, forage, and water *ad libitum*. The animals were reared following the guidelines of the Animal Ethics Committee, Nanjing Agricultural University, China. All experimental protocols were approved by the Science and Technology Agency of Jiangsu Province [approval ID: SYXK (SU) 2010-0005]. All animals were allowed to acclimate for a period of 2 weeks before the trials.

*H. contortus* obtained from Jiangsu Province of China was maintained and propagated in nematode-free lambs as previously described (22). Third-stage larvae (L3) hatched and harvested from fresh goat feces were stored at 4°C for further usage.

### Preparation of rHc8 Antigen and Generation of Polyclonal Antibodies Specific for rHc8

*E. coli* strain expressing rHc8 was constructed with pET28a. The recombinant protein was purified by nickel chelating chromatography through affinity for the hexa-histidine tag. The product of rHc8 antigen was visualized by Coomassie bright blue staining after sodium dodecyl sulfate–polyacrylamide gel electrophoresis (SDS-PAGE) electrophoresis, with a molecular weight of approximately 72 kDa (Supplementary Figure 1). The endotoxin was removed using His Bind® Resin Chromatography kit (Merck, Darmstadt, Germany) and Detoxi-Gel Affinity Pak Prepacked columns (Pierce, Rockford, USA) as described previously (23). The purified proteins were stocked at −70°C for later use.

Healthy local goats (*n* = 3) were used to generate polyclonal antibodies specific for rHc8 (PcAb-rHc8). Briefly, 0.5 mg of purified rHc8 protein formulated with Freund's complete adjuvant (Sigma-Aldrich, St Louis, MO, USA) (1:1 in volume) was subcutaneously injected into goats. The second injection was given to the goats after a 2-week interval, with 0.5 mg of purified rHc8 protein mixed with Freund's incomplete adjuvant (Sigma-Aldrich) (1:1 in volume). The third immunization was performed 1 week after the second immunization. One week after the third immunization, the fourth injection was given to boost the immunized goats. At 1 week after the last injection, the sera containing specific antibodies were collected, purified using Protein G Resin (Genscript, Nanjing, China), and then stored at −30°C for further usage. The specific antibody titer was analyzed by indirect ELISA (1:2^18^ in goat serum) as previously described (24) (Supplementary Figure 2).

### Trial 1: Experimental Design of Passive Immunization With PcAb-rHc8

Goats were randomly allocated into three experimental groups balanced for body weight: Group A, unimmunized and uninfected control goats (*n* = 5); Group B, goats unimmunized but challenged with L3s (*n* = 5); and Group C, goats challenged with L3s (1 week old) and then intravenously immunized with PcAb-rHc8 (*n* = 5). Goats from Groups B and C were infected with 8,000 *H. contortus* L3s by oral gavage on day 1. Goats from Group C were immunized with anti-rHc8-specific serum (5 mg of total IgG for each goat) at day 2 and 3 post-challenge. All goats were sacrificed for necropsies on day 35.

### Trial 2: Experimental Design of Active Immunization With rHc8

Goats were randomly allocated into three experimental groups matched for body weight: Group D, unvaccinated and uninfected control goats (*n* = 5); Group E, unvaccinated but challenged goats (*n* = 5); and Group F, subcutaneously vaccinated with rHc8 and then challenged goats (*n* = 5). Goats of Group F were immunized with 500 μg of rHc8 blended with Freund's complete adjuvant (1:1) (Sigma-Aldrich) on day 0, and 500 μg of rHc8 with Freund's incomplete adjuvant (1:1) (Sigma-Aldrich) on day 15. Goats of Groups E and F were challenged with 8,000 L3s (1 week old) by oral gavage on day 29. Time points for sampling were chosen based on the previous study (25). All goats were sacrificed for necropsies on day 64.

### Parasitological Examinations, Serum, and Mucosal Homogenate Samples

Fresh fecal samples from the rectum of goats were collected on days 18, 20, 22, 24, 26, 28, 30, and 32 in trial 1 and days 50, 52, 54, 56, 58, 60, and 62 in trial 2 to determine FEC following the modified McMaster technique (26). Cumulative FEC (cFEC) was calculated using a linear trapezoidal computational method as previously described (27). In addition, abomasal samples from goats necropsied were taken to classify and enumerate the *H. contortus* according to the techniques described in a previous study (28).

For serum collection, blood samples were taken from the jugular vein using sterile vacuum collective tubes without anticoagulants. The supernatants from serum samples were carefully collected after centrifugation and then stored at −20°C for later use.

To determine antibody responses in the abomasa, mucosal homogenate (MH) samples scraped from the surface of the abomasum were processed as previously described (22). The mucosa samples were homogenized in 3 volumes of cold 0.1 M phosphate-buffered saline (PBS) (pH 7.4) containing 5 mM ethylenediaminetetraacetic acid (EDTA) and 5 mM phenylmethylsulfonyl fluoride (PMSF) overnight at 4°C, followed by centrifugation at 10,000 × *g* for 20 min. The supernatants were collected and then stored at −20°C for later usage.

### Detection of Goat Serum Antibody Levels Specific for rHc8

The rHc8-specific antibody levels were ascertained by ELISA. The appropriate rHc8 concentration (250 ng/μl) and the optimal dilution of goat serum (1: 200) were determined by chessboard titration. In brief, 100 μl of rHc8 diluted in carbonate coating buffer (0.05 M, pH 9.6) was coated on 96-well microliter plates at 4°C overnight. Skim milk (5%) in PBS containing 0.5% Tween 20 (PBST) was used to block the plates at 37°C for 1 h. As to the primary antibody, diluted goat serum in PBST was incubated at 37°C for 1 h, followed by washing three times. The horseradish peroxidase (HRP)-conjugated rabbit anti-goat IgG (H + L) (Thermo Scientific, Waltham, MA, USA) was then used in l:5,000 dilution at 37°C for 1 h. After five washes, the colorimetric reaction was initiated with 3, 3′, 5, 5′-tetramethylbenzidine (TMB) substrate. The substrate reactions were terminated with 2 M sulfuric acid, and absorbance values at 450 nm (OD450) were measured by a microplate reader (Thermo Scientific).

### Measurement of Antibody Levels in MH Samples by ELISA

Goat IgG, IgA, and IgE ELISA Quantization Kits (MLBIO, Shanghai, China) were used to measure antibody levels of each group according to the manufacturer's specifications.

### Complete Blood Count

Blood samples were harvested from jugular vein using sterile vacuum tubes with anticoagulant EDTA-K2, and blood count examination was performed using the whole blood by a blood test instrument (BC5000-Vet blood cell analyzer, Mindray, Shenzhen, China).

### Statistical Analysis

GraphPad Premier 8.0 software package (GraphPad Prism, San Diego, CA, USA) was used for the statistical analysis. For the selection of the appropriate tests, normality distribution determined by the Shapiro–Wilk test were assessed to ensure the correctness of conclusions. Two-tailed, unpaired Student's *t*-test was used for cFEC. Comparisons of worm burden data were performed using Mann–Whitney *U*-tests. Repeated measures (RM) analysis of variance (ANOVA) methods with Tamhane's T2 multiple comparison test were conducted for antibody levels and hematological parameters. Data were represented as mean ± the standard error of mean (SEM). *p* < 0.05 were considered significant.

## Results

### FEC, cFEC, and Worm Burdens of Trial 1 (Groups A–C)

**Figure 1:**
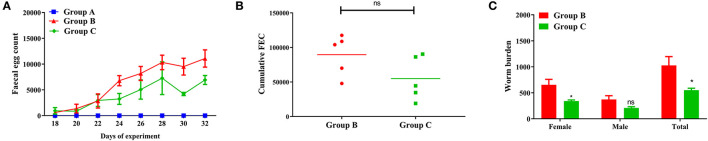
Dynamics of egg outputs and statistics of abomasum worm burdens in trial 1. Group A, unchallenged goats; Group B, goats challenged with L3s at day 1; Group C, goats challenged with L3s at day 1 and then immunized by PcAb-rHc8 antibodies at days 2 and 3 post challenge. **(A)** FEC are presented as mean ± SEM. **(B)** Mean cFEC values presented the data of each goat in each group. **(C)** Worm burdens are presented as mean ± SEM (*p < 0.05, ns, non-significant vs. infection group), and a capped line designate significance between two groups (ns, non-significant).

FEC for passive immunization in trial 1 are plotted in Figure 1A. The FEC of goats passively immunized with PcAb-rHc8 (Group C) were significantly lower than those from infected controls (Group B). The overall mean cFEC per animal was reduced by 39% in trial 1 compared with infected controls (*p* > 0.05; Figure 1B).

As shown in Figure 1C, goats in Group C immunized with PcAb-rHc8 had decreased total worm burdens compared to the infected controls, with the reduction rate of 46% (*p* < 0.05). In addition, both female and total worm counts in the challenge control group were significantly higher than the immunization group (*p* < 0.05 for both).

### MH Antibodies Levels in Trial 1

**Figure 2:**
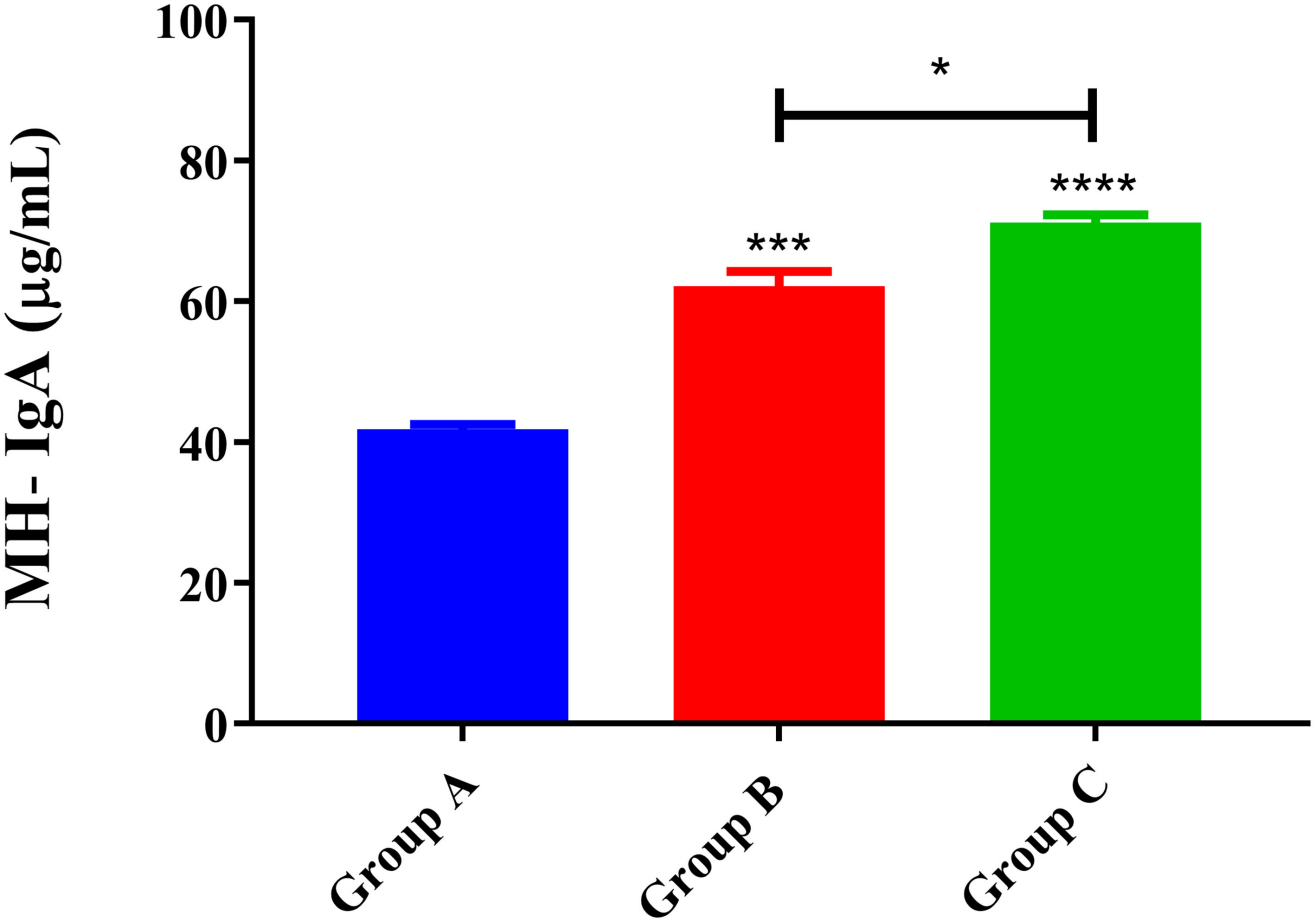
MH-IgA contents of abomasum measured in trial 1. Group A, unchallenged goats; Group B, goats challenged with L3s at day 1; Group C, goats challenged with L3s at day 1 and then immunized by PcAb-rHc8 antibodies at days 2 and 3 post-challenge. Data are expressed as mean ± SEM. ***p < 0.001, ****p < 0.0001 vs. blank group and a capped line designates two groups that differ significantly (*p < 0.05).

The MH-IgA levels of goats in trial 1 are presented in Figure 2. The results showed that abomasum MH-IgA levels in both Group C (PcAb*-*rHc8 immunization goats, 71.2 ± 1.15) and Group B (infected controls, 62.1 ± 2.18) were significantly higher than those in Group A (blank controls, 41.8 ± 0.730) (*p* < 0.001 and *p* < 0.0001, respectively). Specifically, a statistically significant difference in MH-IgA levels was also observed between Groups B and C (*p* < 0.05) in trial 1. However, MH-IgG and IgE values did not differ significantly between groups (data not shown).

### Specific Antibody Detection From Goat Sera in Trial 1

**Figure 3:**
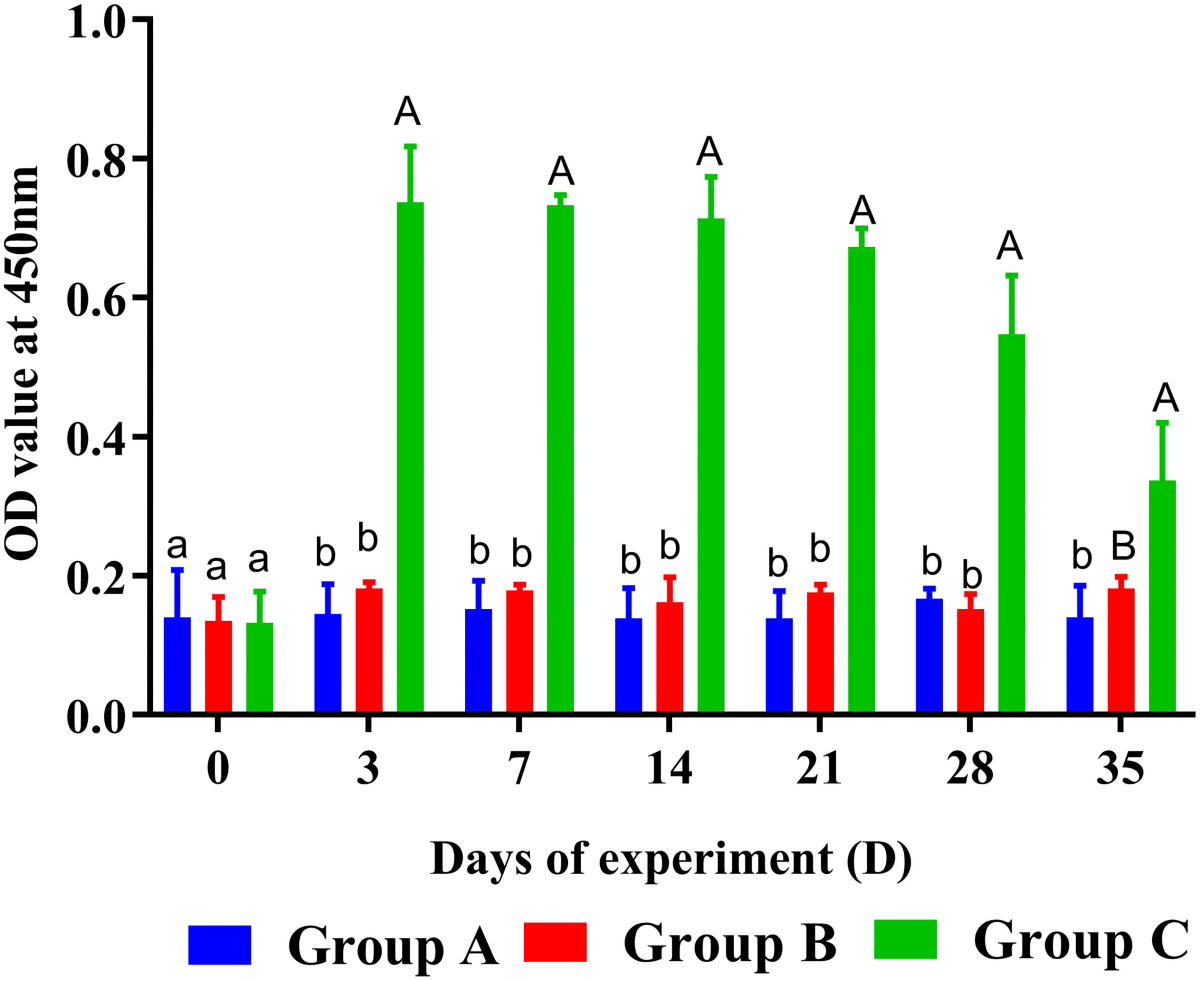
Dynamics of rHc8-specific IgG in sera of trial 1. Group A, unchallenged goats; Group B, goats challenged with L3s at day 1; Group C, goats challenged with L3s at day 1 and then immunized by PcAb-rHc8 antibodies at days 2 and 3 post challenge. Data were expressed as mean ± SEM. Same letter indicates nonsignificant difference (*p* > 0.05). The different lowercase letters indicate significantly different values at *p* < 0.05. Different uppercase and lowercase letters indicate significantly different values *p* < 0.01.

The levels of specific anti-rHc8 IgG were estimated by ELISA. They were kept at much higher levels in PcAb-rHc8 immunization goats compared with the two control groups from day 3 to day 28 (Figure 3). Besides, goats in the infected group also had slightly higher antibody levels than unimmunized and uninfected control goats (*p* > 0.05).

### Full Blood Count Analysis in Trial 1

**Figure 4:**
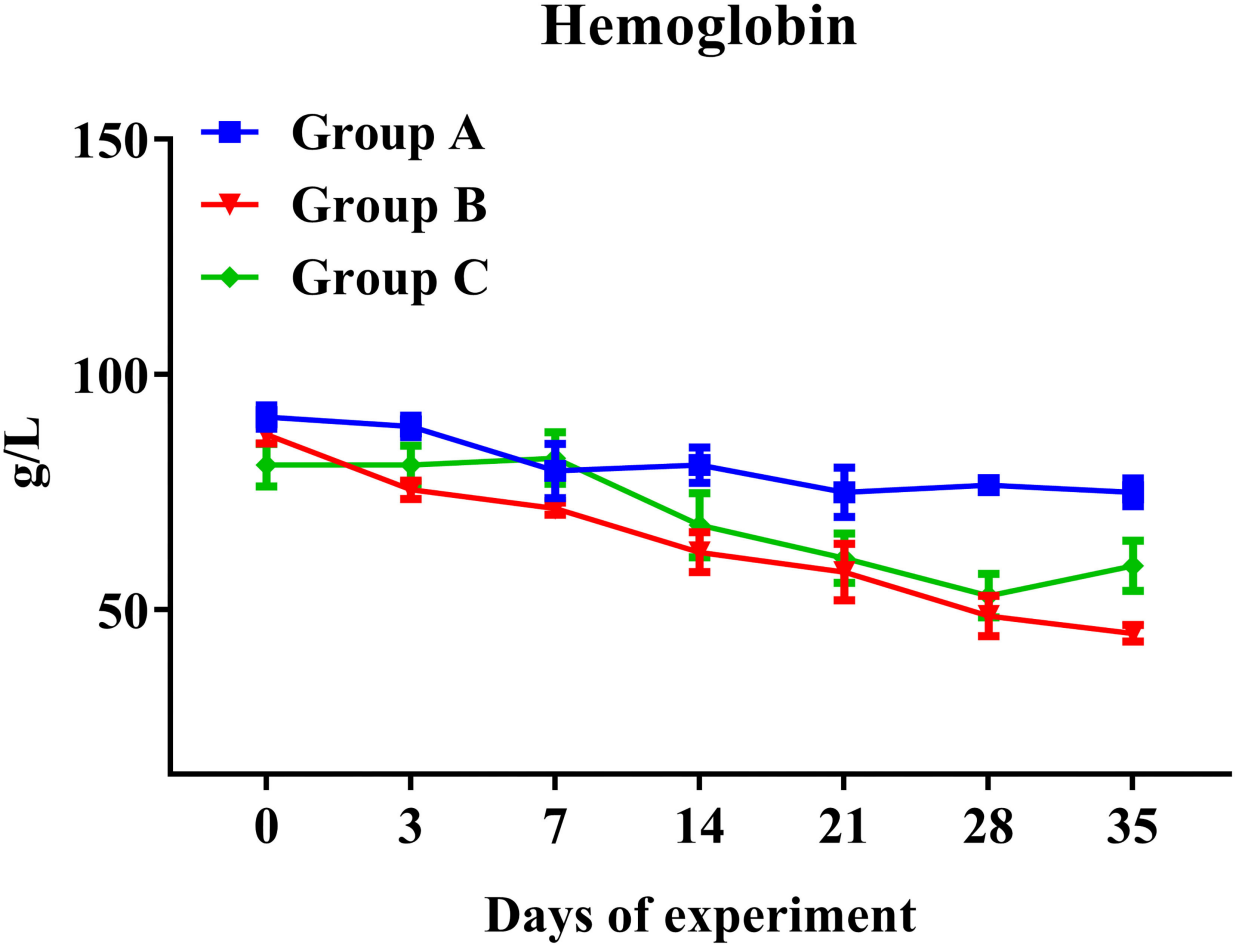
Hemoglobin levels in trial 1. Group A, unchallenged goats; Group B, goats challenged with L3s at day 1; Group C, goats challenged with L3s at day 1 and then immunized by PcAb-rHc8 antibodies at days 2 and 3 post challenge. Hemoglobin (HGB) contents of blood samples were determined. Data are shown as means ± SEM.

As expected, the concentration of hemoglobin presented a significantly decreased trend in all challenged goats (Groups B and C), while it remained stable in unchallenged controls (Group A) (Figure 4). In addition, hemoglobin levels of Groups B and C showed broadly similar traits. Hemoglobin levels of PcAb-rHc8 immunized goats appeared to be higher than those of challenged controls at day 35, but no significant difference was observed (*p* > 0.05; Figure 4). However, as for other hematological parameters, no notable changes were observed in red blood cells, hematocrit, or eosinophils in all groups (data not shown).

### FEC, cFEC, and Worm Burdens in Trial 2 (Groups D–F)

**Figure 5:**
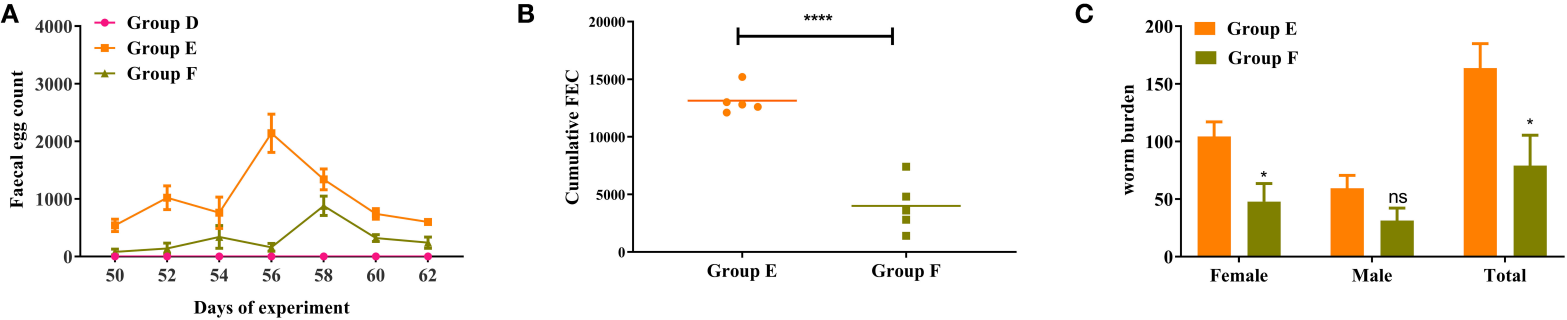
Dynamics of egg outputs and statistics of abomasum worm burdens in trial 2. Group D, unchallenged goats; Group E, goats challenged with L3s; Group F, goats vaccinated by rHc8 antigen mixed with Freund's adjuvants (at day 0 and 15), and challenged with L3s (at day 29). **(A)** FEC are presented as mean ± SEM. **(B)** Mean cFEC values present the data of each goat in each group. **(C)** Worm burdens are presented as mean ± SEM. *p < 0.05, **p < 0.01; ns, non-significant vs. infection group, a capped line designates significance between two groups (****p < 0.0001).

The dynamics of FEC for active immunization trial is graphically displayed in Figure 5A. The FEC in goats vaccinated with rHc8 antigen (Group F) were lower than those in the infected controls (Group E) throughout trial 2. As indicated in Figure 5B, a significant 70% reduction from overall mean cFEC per animal was observed (*p* < 0.0001).

Goats vaccinated with rHc8 in Group F had a significant decrease in total worm burdens compared to the infected controls (Group E), showing a reduction rate of 55% (*p* < 0.05; Figure 5C). In contrast, no significant differences in male burdens between these two groups were observed (Figure 5C). In addition, a significant reduction in female worms was found in the vaccinated group compared with the challenged control group (*p* < 0.05; Figure 5C).

### MH-IgA Levels in Trial 2

**Figure 6:**
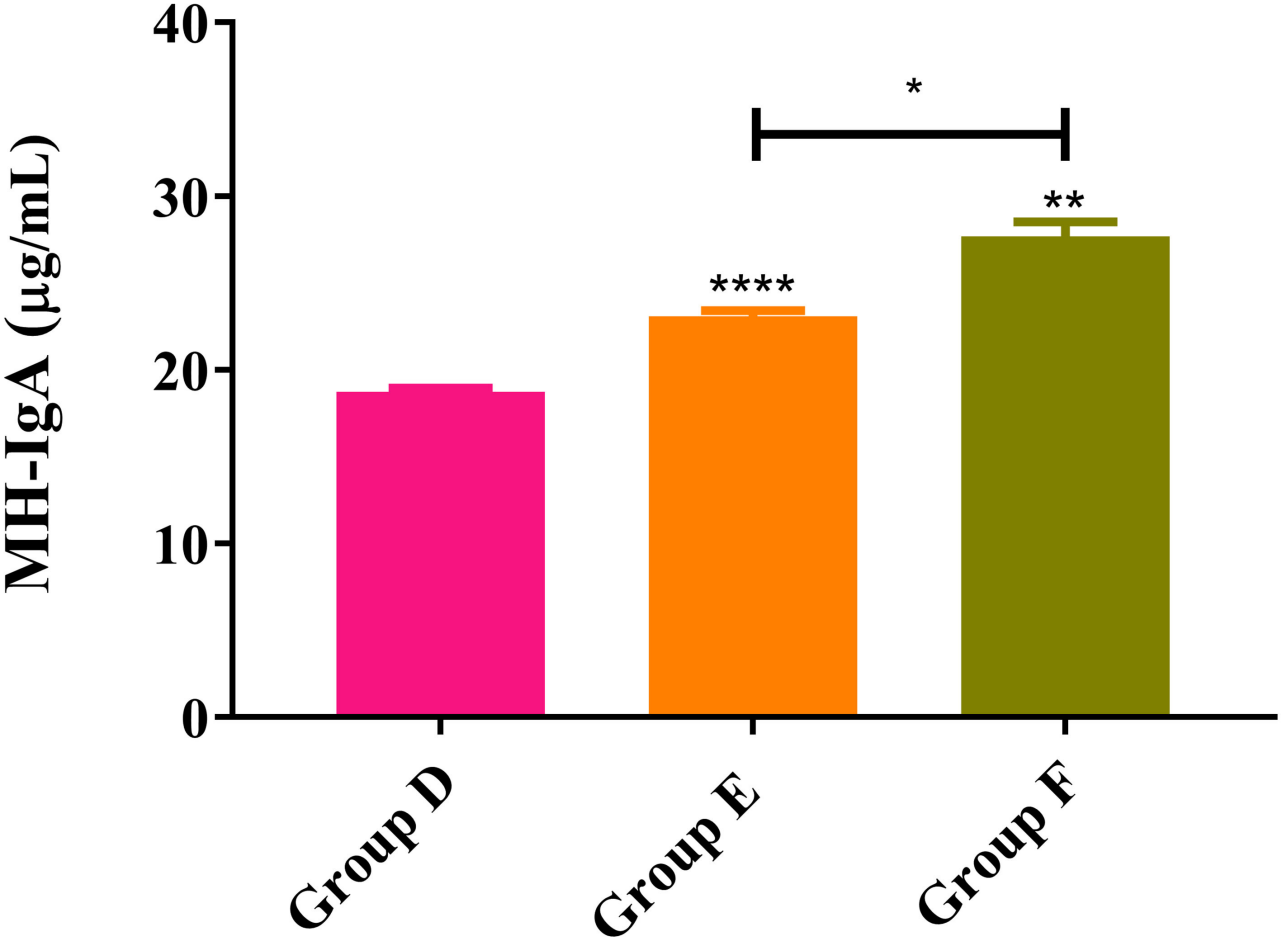
MH-IgA contents detected in abomasum of trial 2. Group D, unchallenged goats; Group E, goats challenged with L3s; Group F, goats vaccinated by rHc8 antigen mixed with Freund's adjuvants (at day 0 and 15), and challenged with L3s (at day 29). Data were expressed as mean ± SEM. **p < 0.01, ****p < 0.0001 vs. blank group and a capped line designates two groups that differ significantly (*p < 0.05).

As shown in Figure 6, ELISA results from trial 2 revealed that the MH-IgA levels of both rHc8-vaccinated goats (Group F: 26.6 ± 1.25) and infected controls (Group E: 23.1 ± 0.330) were significantly higher than unimmunized and uninfected control goats (Group D: 18.7 ± 0.230) (*p* < 0.0001 and *p* < 0.01, respectively). Additionally, MH-IgA levels of vaccinated goats in Group F were significantly higher than the challenged controls in Group E (*p* < 0.05; Figure 6). Similar to trial 1, significant differences were observed in the levels MH-IgG or MH-IgE across all groups (data not shown).

### Specific Antibody Detection From Goat Sera in Trial 2

**Figure 7:**
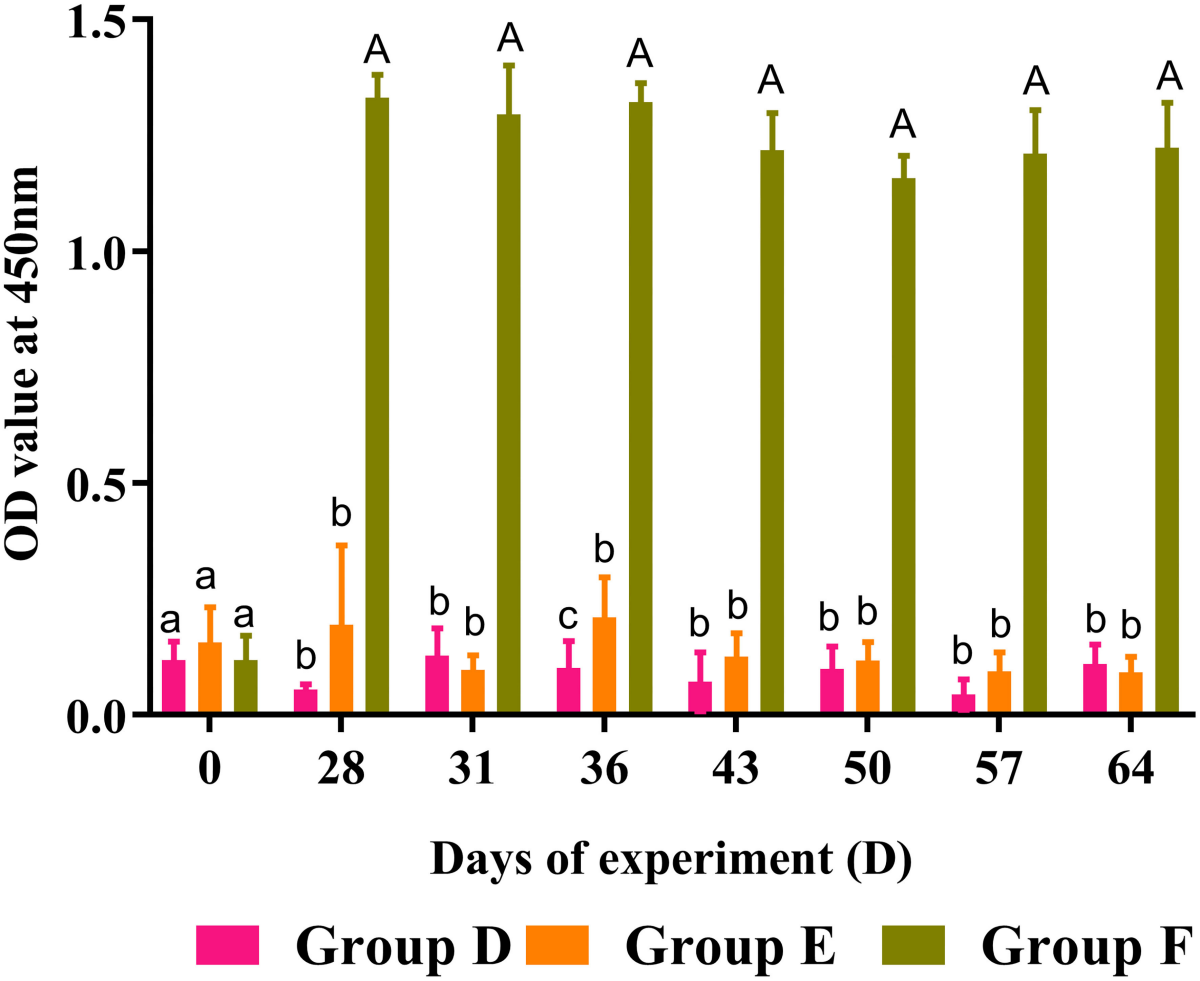
Dynamics of rHc8-specific IgG in sera of trial 2. Group D, unchallenged goats; Group E, goats challenged with L3s; Group F, goats vaccinated by rHc8 antigen mixed with Freund's adjuvants (at day 0 and 15), and challenged with L3s (at day 29). Data are presented as mean ± SEM. Same letter indicates nonsignificant difference (*p* > 0.05). The different lowercase letters indicate significantly different values at *p* < 0.05. Different between uppercase and lowercase letters indicate significantly different values *p* < 0.01.

Circulating anti-rHc8 IgG levels in goat sera were detected by ELISA, and the results are demonstrated in Figure 7. Goats vaccinated with rHc8 in Group F displayed robust and sustained immune responses throughout trial 2, demonstrated by the significantly higher titers of specific anti-rHc8 IgG compared with both blank and challenged controls from day 28 (the second immunization) till the end of the experiment (*p* < 0.01 for all time points). In contrast, there were no significant differences in antibody levels between the infected controls (Group E) and the blank controls (Group D).

### Full Blood Count Analysis in Trial 2

**Figure 8:**
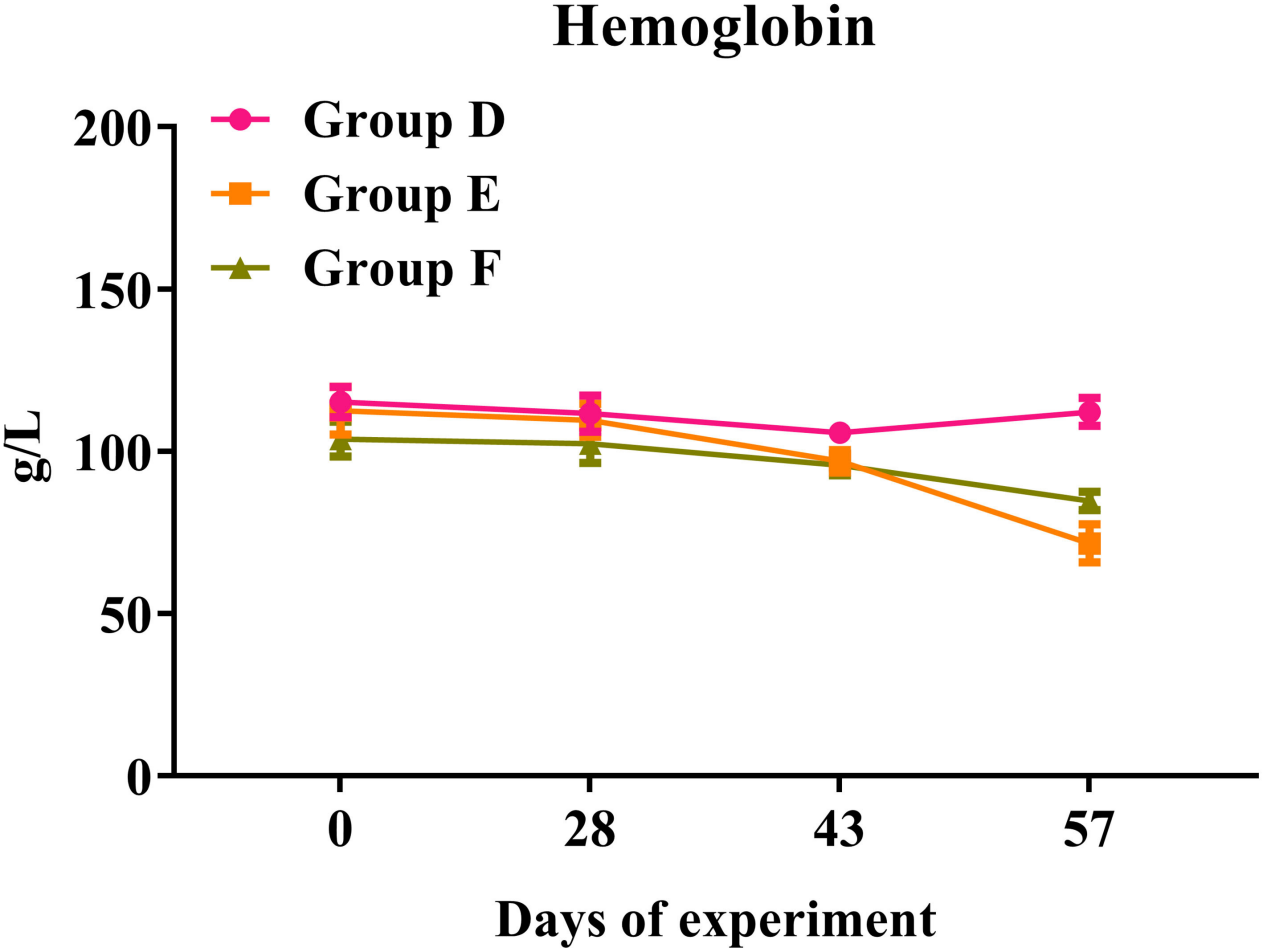
Hemoglobin levels in trial 2. Group D, unchallenged goats; Group E, goats challenged with L3s; Group F, goats vaccinated by rHc8 antigen mixed with Freund's adjuvants (at day 0 and 15), and challenged with L3s (at day 29). Hemoglobin (HGB) contents of blood samples were determined. Data are shown as means ± SEM.

In trial 2, both challenged groups presented a significant reduction in hemoglobin levels over the time course of the infection (Figure 8). Compared with the infected controls in Group E, rHc8-vaccinated goats in Group F showed a slight decrease in hemoglobin levels. By the way, rHc8-vaccinated goats in Group F seemed to have a slightly higher level than the infected controls in Group E on day 57. However, the difference between these two groups was not statistically significant (*p* > 0.05; Figure 8). Similar to trial 1, there were no significant differences in the levels of red blood cells, hematocrit or eosinophils across all groups (data not shown).

## Discussion

With regards to immune regulation, IL-2 displays an indispensable role as an immune regulatory factor, which could promote T-cell proliferation and survival *in vitro* (29, 30). It is noteworthy that IL-2 as an adjuvant could improve the efficacy of the DNA vaccination encoding H11 protein against *H. contortus* infection (28). Accumulating studies reveal that massive ES proteins produced by *H. contortus* result in a complex and sophisticated immune response in the host during infection (8, 9, 31). In our preliminary work, Hc8 protein was identified from HcESPs and interacted with host IL-2. Besides, Hc8 acted as an antagonist that interfered with the biological activities of IL-2, along with associated signaling pathways (20). Based on these findings, employing anti-rHc8 antibodies that specifically bind to Hc8 *in vivo* might help to retrieve the function of IL-2 and host protective immunity against *H. contortus*. In this research, immunization with PcAb-Hc8 antibodies conferred partial but statistically significant protection to challenged goats, showing a 39% reduction in egg shedding and a 46% reduction in worm burden compared with challenged controls. These results indicated that anti-Hc8 antibodies seemed to act effectively against *H. contortus* infections to some extent, although future efforts are needed to validate the protective effects in field trials.

Vaccines could reduce the risk of drenching and pasture contamination, which may, in turn, lessen the chance of repeated infections of *H. contortus* in susceptible animals. Unfortunately, to date, there are few descriptions of viable vaccines being successful for *H. contortus*. The only commercialized vaccine, Barbervax encompassing various native antigens, attained an excellent performance in sheep. Besides, for more susceptible goats, studies demonstrated that the average reductions of FEC were 69.8 ± 11.7% and 57.4 ± 17.6% for the Anglo Nubians and Saanens, respectively (32). In addition to native antigen preparations, recombinant subunit vaccines warrant much exploration given their commercial applicability and reliable and reproducible efficacy. Numerous recombinant *H. contortus* antigens were tested either in laboratory or field trials, but few of them have been reported to be effective. To develop an effective vaccine against *H. contortus*, the top priority was to reduce egg shedding and worm burdens in infected hosts (33). In this study, the data in trial 2 demonstrated that vaccination with rHc8 antigen could induce significant protection to the hosts, showing a 70% reduction in egg excretion and a 55% reduction in worm burden when compared with challenged controls. It is likely that the immune responses induced by the administration of rHc8 antigen might effectively reduce egg-laying capacity and worm burdens. In addition, rHc8, as a novel protective antigen, may contribute to the development of a cocktail vaccine encompassing multiple recombinant proteins against *H. contortus*, given the success of the recombinant cocktail vaccine for *Teladorsagia circumcincta* (34, 35).

As a regulatory factor, the correlation of IgA with host immunity against *H. contortus* were not fully understood. However, a spectrum of reports shows that MH-IgA appeared to engage in host immune responses and played essential roles in parasite invasion and expulsion associated with FECs (25, 36, 37). Sun et al. revealed that the relationship between MH-IgA and abomasum worm burdens was remarkably negatively correlated based on Spearman's rank correlation coefficient (37). This study showed that MH-IgA levels in response to active and passive immunization in challenged goats were higher than those in the blank controls in both trials 1 and 2, suggesting the essential roles of host IgA in mucosal immunity against *H. contortus*. In addition, regardless of given antibodies or recombinant proteins, mucosal IgA levels in immunized goats were higher than the challenged controls in both trials. A previous study observed significantly increased mucosal IgA levels in goats boosted with IL-2 as an adjuvant, suggesting the positive correlation of host IL-2 levels with mucosal IgA production (28). The possible reason for increased mucosal IgA levels in immunized goats could be that anti-Hc8 IgG blocked the antagonistic effects of Hc8 on host IL-2, and the latter might magnify mucosal IgA generation and mucosal immune responses. However, the detailed mechanism merits further investigation. Additionally, ELISA results showed that Hc8-specific IgG maintained high levels throughout both trials 1 and 2. High circulating antibody levels were considered to be critical for offering a long period of protection for animals (38). All these data indicated that MH-IgA and antigen-specific IgG in response to immunizations could function as important indicators and contribute to host immune protection against *H. contortus*.

Hemoglobin levels were considered an essential hematological parameter for the diagnosis of anemia and a hallmark for the assessment of severity in haemonchosis. Previous studies indicated that hemoglobin level was negatively correlated with adult worm counts and egg shedding (39, 40). However, Yanming et al. reached a contradictory conclusion that there was no significant association between hemoglobin values and worm burdens (37). Although actively or passively immunized goats appeared to show higher hemoglobin levels than the challenged controls at certain time points in both trials, no significant differences were observed in this study. In addition, the hemoglobin levels in challenged goats showed consistently downward trends over the time course of infections, but there was no significance when compared to the unchallenged controls in both trials 1 and 2. Possibly, adult worms were present for a too-short time period to influence hemoglobin levels significantly.

## Conclusion

In this study, we evaluated the immune protection efficacies of PcAb-Hc8 antibodies and Freund's adjuvanted rHc8 antigens in an experimental infection model. Both eggs shedding and worm burdens were significantly decreased in the passive or active immunization trials. Partial protections were observed when challenged goats were administrated with PcAb-Hc8 antibodies, while active immunization with Freund's adjuvanted Hc8 antigens resulted in strong protection efficacy. Although the specific mechanism still requires further investigation, this study indicated the possibility of rHc8 antigens as a potential vaccine candidate. In addition, rHc8 antigen merits further investigation for haemonchosis prevention and control in the setting of field trials.

## Data Availability Statement

The raw data supporting the conclusions of this article will be made available by the authors, without undue reservation.

## Ethics Statement

The animal study was reviewed and approved by Science and Technology Agency of Jiangsu Province.

## Author Contributions

XL, MLu, CL, and XT: conception or design of this study. XL, LX, RY, XS, and CL: direction and supervision. XT: data analysis and drafting this article. XT, CL, YB, KA, YZ, and MLi: material acquisition. All authors read and approved the final manuscript.

## Funding

This work was funded by grants from the National Key Research and Development Program of China (Grant No. 2017YFD0501200) and the policy guidance project of Jiangsu Province for international scientific and technological cooperation (Grant No. BZ2019013).

## Conflict of Interest

The authors declare that the research was conducted in the absence of any commercial or financial relationships that could be construed as a potential conflict of interest.

## Publisher's Note

All claims expressed in this article are solely those of the authors and do not necessarily represent those of their affiliated organizations, or those of the publisher, the editors and the reviewers. Any product that may be evaluated in this article, or claim that may be made by its manufacturer, is not guaranteed or endorsed by the publisher.

## Supplementary Material

Supplementary Figure 1 Electrophoresis analysis (SDS–PAGE) of the purification of rHc8 protein. Protein sample was clearly displayed by Coomassie brilliant blue R250 on 12% of polyacrylamide gel. M showed standard protein ladder; Lane 1 showed purified recombinant Hc8.

Supplementary Figure 2 ELISA analysis of specific antibody titer anti-rHc8 protein. Normal goat serums were utilized as negative controls. The cut-off values (0.41) are shown with a dotted line.

Supplementary Figure 3 Original picture of Supplementary Figure 1.

Supplementary Table 1 Adult worm burden at necropsy and Fecal egg counts (FEC).
