# Supplementary figures and images for: Immunization With Recombinant Haemonchus contortus Y75B8A.8 Partially Protects Local Crossbred Female Goats From Haemonchus contortus Infection
Source: Front Vet Sci. 2022 Apr 4;9:765700. doi: 10.3389/fvets.2022.765700 (PMC9014092; doi:10.3389/fvets.2022.765700)

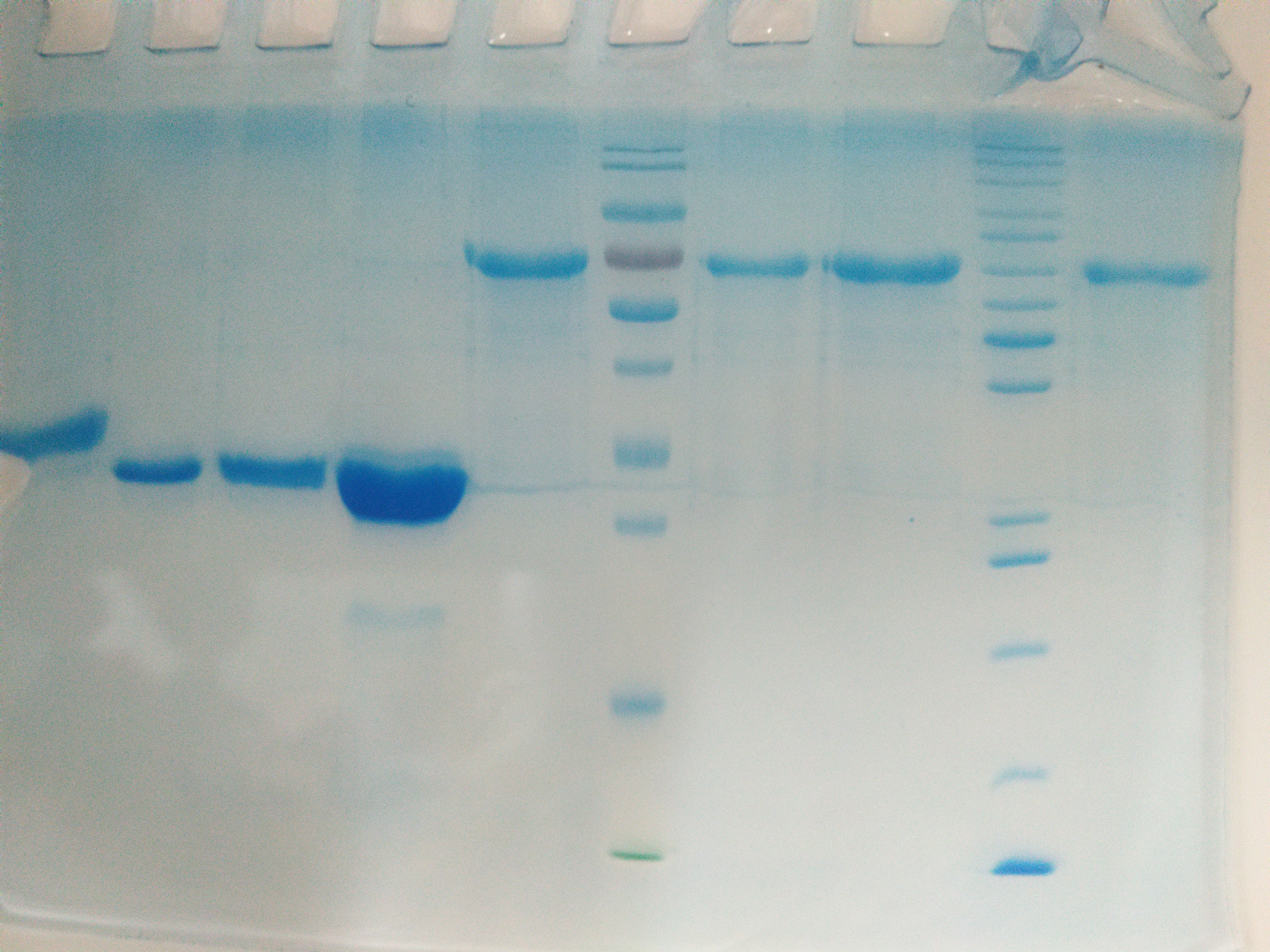

Supplement: Supplementary Figure 1 — Electrophoresis analysis (SDS–PAGE) of the purification of rHc8 protein. Protein sample was clearly displayed by Coomassie brilliant blue R250 on 12% of polyacrylamide gel. M showed standard protein ladder; Lane 1 showed purified recombinant Hc8. [file Image_1.JPEG]

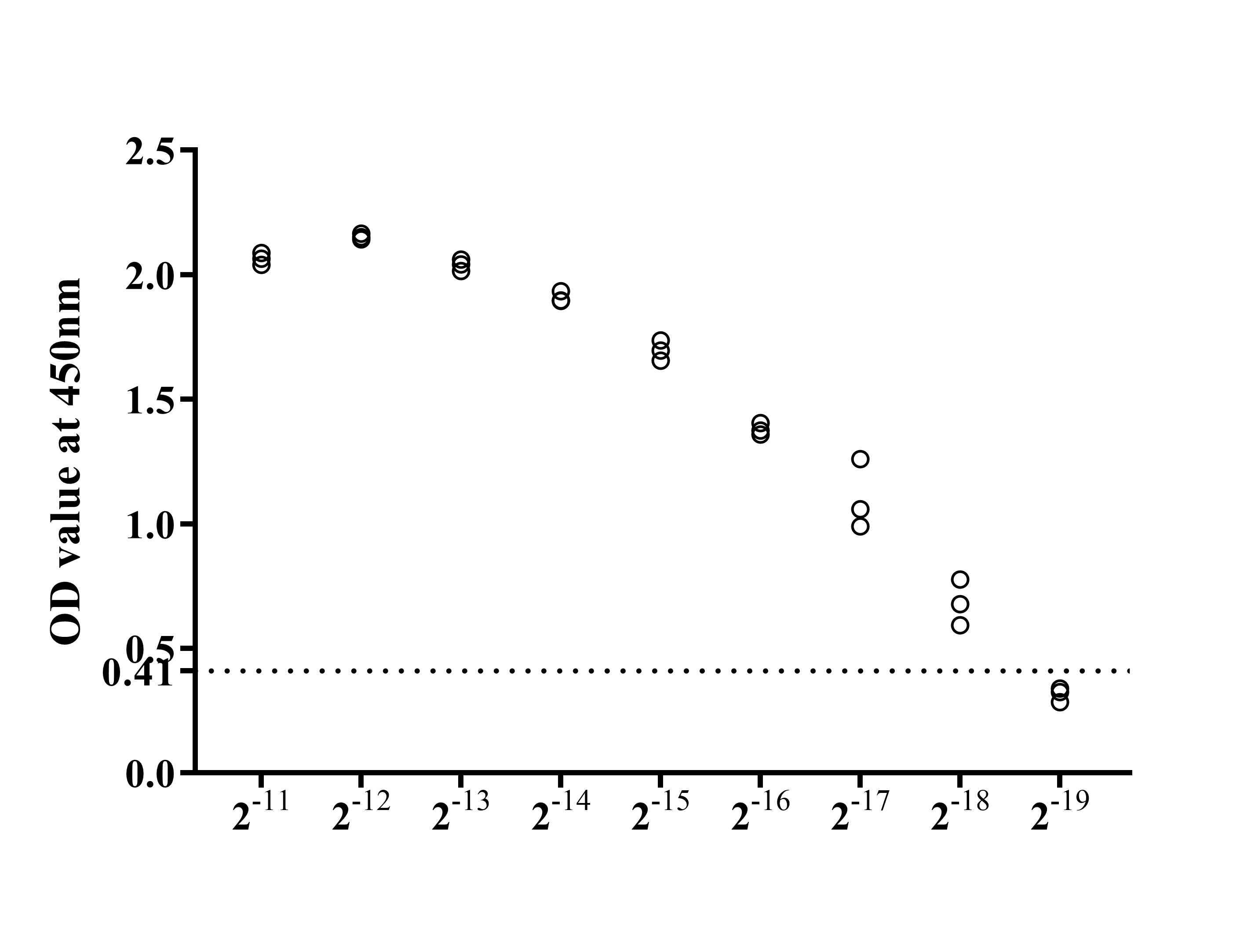

Supplement: Supplementary Figure 2 — ELISA analysis of specific antibody titer anti-rHc8 protein. Normal goat serums were utilized as negative controls. The cut-off values (0.41) are shown with a dotted line. [file Image_2.TIF]

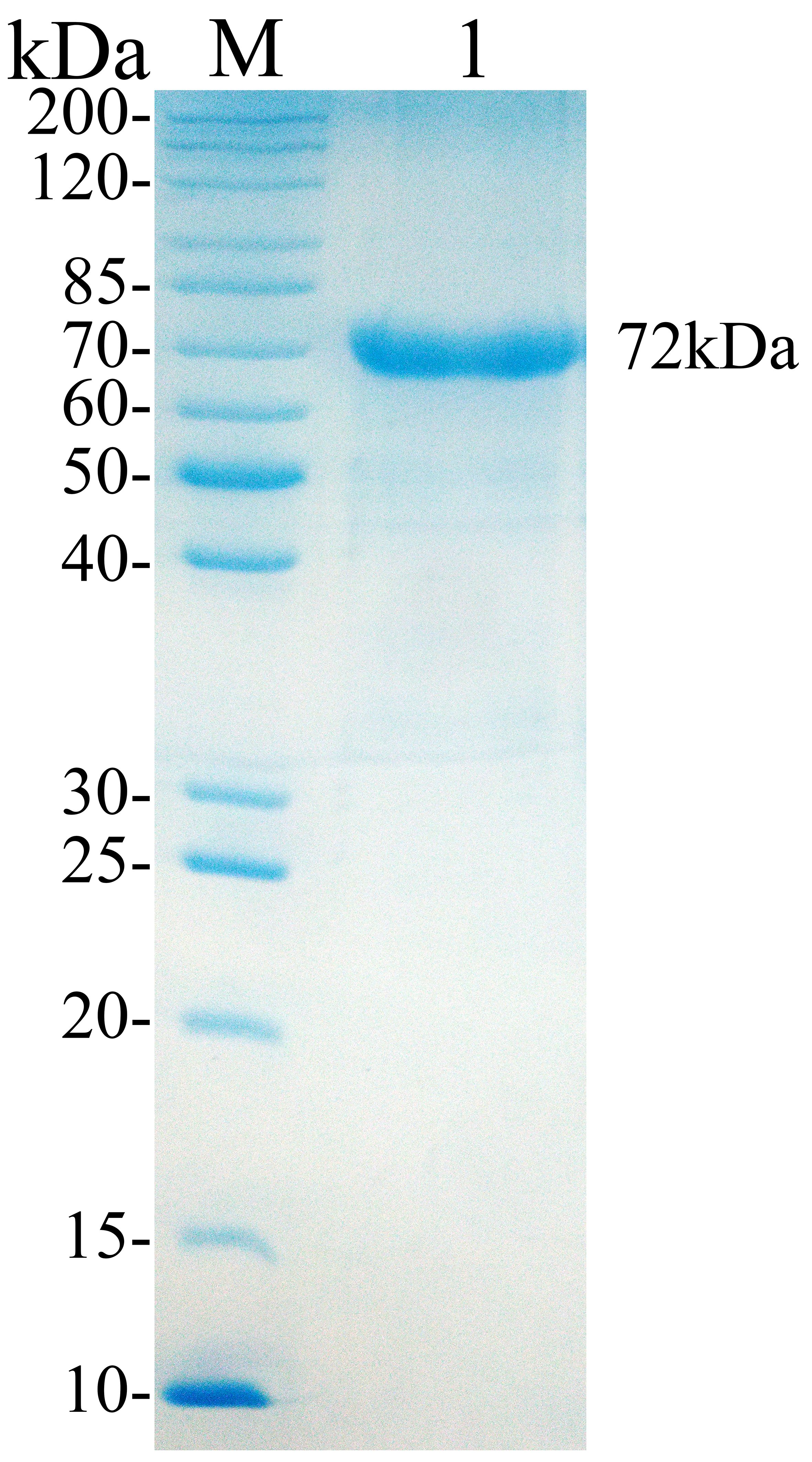

Supplement: Supplementary Figure 3 — Original picture of Supplementary Figure 1. [file Image_3.TIF]
