# Supplementary material for: Immunization With Recombinant Haemonchus contortus Y75B8A.8 Partially Protects Local Crossbred Female Goats From Haemonchus contortus Infection
Source: Front Vet Sci. 2022 Apr 4;9:765700. doi: 10.3389/fvets.2022.765700 (PMC9014092; doi:10.3389/fvets.2022.765700)
Supplement: Supplementary Table 1 — Adult worm burden at necropsy and Fecal egg counts (FEC). [file Table_1.DOCX]

Table S1. Adult worm burden at necropsy and Fecal egg counts (FEC)

| Group | Adult wroms (Mean±SEM) | | | Days of experiment | FEC  (Mean±SEM) |
| --- | --- | --- | --- | --- | --- |
|  | Female | Male | Total |  |  |
| A | 0 | 0 | 0 | 18 | 0 |
|  |  |  |  | 20 | 0 |
|  |  |  |  | 22 | 0 |
|  |  |  |  | 24 | 0 |
|  |  |  |  | 26 | 0 |
|  |  |  |  | 28 | 0 |
|  |  |  |  | 30 | 0 |
|  |  |  |  | 32 | 0 |
| B | 655±104.2 | 372±71.5 | 1027±170.0 | 18 | 620±310.5 |
|  |  |  |  | 20 | 1360±847.7 |
|  |  |  |  | 22 | 2840±1371.3 |
|  |  |  |  | 24 | 6780±979.5 |
|  |  |  |  | 26 | 8180±1351.8 |
|  |  |  |  | 28 | 10520±1418.6 |
|  |  |  |  | 30 | 9500±1627.3 |
|  |  |  |  | 32 | 11080±1678.5 |
| C | 342±20.4 | 209±24.2 | 551±36.4 | 18 | 940±624.2 |
|  |  |  |  | 20 | 850±308.2 |
|  |  |  |  | 22 | 2940±1148.3 |
|  |  |  |  | 24 | 3240±1061.4 |
|  |  |  |  | 26 | 5060±1866.7 |
|  |  |  |  | 28 | 7260±3171.8 |
|  |  |  |  | 30 | 4200±308.2 |
|  |  |  |  | 32 | 6920±869.7 |
| D | 0 | 0 | 0 | 50 | 0 |
|  |  |  |  | 52 | 0 |
|  |  |  |  | 54 | 0 |
|  |  |  |  | 56 | 0 |
|  |  |  |  | 58 | 0 |
|  |  |  |  | 60 | 0 |
|  |  |  |  | 62 | 0 |
| E | 104±12.7 | 59±11.0 | 164±21.2 | 50 | 540±107.7 |
|  |  |  |  | 52 | 1020±205.9 |
|  |  |  |  | 54 | 760±273.1 |
|  |  |  |  | 56 | 2140±331.1 |
|  |  |  |  | 58 | 1340±180.6 |
|  |  |  |  | 60 | 740±92.7 |
|  |  |  |  | 62 | 600±54.8 |

| F | 48±15.6 | 31±11.0 | 79±26.4 | 50 | 80±49.0 |
| --- | --- | --- | --- | --- | --- |
|  |  |  |  | 52 | 140±92.7 |
|  |  |  |  | 54 | 340±196.5 |
|  |  |  |  | 56 | 160±67.8 |
|  |  |  |  | 58 | 880±168.5 |
|  |  |  |  | 60 | 320±58.3 |
|  |  |  |  | 62 | 240±98.0 |
